# Environmental exposure to lead and cadmium and hearing loss in Chinese adults: A case-control study

**DOI:** 10.1371/journal.pone.0233165

**Published:** 2020-05-20

**Authors:** Da-Hui Wang, Hui Xu, Yi-Hua Zheng, Dong-Sheng Gu, Ya-Jun Zhu, Ying Ren, Shi-Chang Wang, Lei Yang, Liang-Wen Xu

**Affiliations:** 1 Medical School, Hangzhou Normal University, Hangzhou, Zhejiang, PR China; 2 Jiangshan People's Hospital, Jiangshan, Zhejiang, PR China; 3 Hospital of Zhejiang Provincial Headquarters of the Chinese People's Armed Police Force, Jiaxing, Zhejiang, PR China; Stony Brook University, Graduate Program in Public Health, UNITED STATES

## Abstract

Hearing loss is the second most common nonfatal problem affecting the Chinese population. Historical studies have suggested an association between exposure to heavy metals, such as cadmium and lead, and hearing loss. Few studies have investigated this relationship in the general population in China. We conducted a case-control study with 1008 pairs of participants from a cross-sectional epidemiological survey conducted in Zhejiang Province. A self-designed questionnaire was adopted to collect information on demographics, chronic diseases, lifestyles and environmental noise. Pure-tone averages of hearing thresholds at frequencies of 0.5, 1, 2, and 4 kHz were computed. Blood lead and cadmium levels were analyzed with an atomic absorption spectrometer. After adjusting for all other potential confounding factors, compared with the lowest blood cadmium quartile (0.00–0.53 μg/L), blood cadmium quartile 2 (0.54–0.92 μg/L), quartile 3 (0.93–1.62 μg/L) and quartile 4 (1.63–57.81 μg/L) exhibited significantly elevated risks for hearing loss, with odds ratios of 1.932 (95% CI: 1.356–2.751), 2.036 (95% CI: 1.423–2.914) and 1.495 (95% CI: 1.048–2.133), respectively (P-trend<0.001). However, an association of lead with hearing loss was not found. Young age (less than 60 years), male sex and current smoking were associated with increased blood cadmium concentration. Additionally, a positive association between blood cadmium and lead concentrations was found. Therefore, we conclude that exposure to environmental cadmium may be a risk factor for hearing loss among the general population in China.

## Introduction

Hearing loss is a critical global public health issue [1]. Currently, 5% of the adult population in the world has hearing loss, and its prevalence increases to 30% when individuals reach 65 years old [2]. Hearing loss may lead to negative consequences, such as disturbed communication, feelings of loneliness and frustration [3], reduced quality of life, and financial loss [4].

Ototoxicants, such as heavy metals, organochlorines and drugs, are substances that can induce damage to the structures and nervous system of the inner ear [5]. Historically, the ototoxic effects of heavy metals have been overlapped with the dominant effect of noise [6]. Environmental cadmium and lead are the most common toxic heavy metals [7, 8]. Cadmium is widely used in the production of batteries, solar panels, pigments, and plastic stabilizers [7]. Lead exposure among the general population mainly results from gasoline, paint, solder and pipes while occupational exposure results from battery manufacturing, steel welding or cutting operations, printing, and construction [9]. Lead and cadmium can reduce blood flow and lipid peroxidation in the cochlea, result in latency in auditory nerve conduction and elevate auditory thresholds [10, 11]. Additionally, lead exposure can cause the degeneration of receptor cells in the inner ear [12], while cadmium induces apoptosis of receptor cells in the inner ear and changes their arrangement [7] [13].

To date, no scientific studies have shown beneficial effects of lead or cadmium on human physiological health, which means that their ideal content in the human body is zero owing to their harmfulness [14]. Therefore, studying the effects of low levels of these heavy metals on human health has become meaningful in both occupational workers and the general populations. In a study conducted in 609 lead-acid battery factory workers, blood lead (>10 μg/dL) was significantly correlated with high-frequency hearing loss, with a 3.98-times higher risk in the highest quartile compared to the lowest quartile after adjusting for potential confounders including noise exposure [15]. Hwang et al. found that even a low level of blood lead (7 μg/dL) exposure would enhance noise-induced hearing loss in 412 steel workers [16]. Choi et al. analyzed the effects of lead and cadmium exposure on hearing loss in 3,698 American adults using the 1999–2004 National Health and Nutrition Examination Survey. They found that low exposure to cadmium and lead can cause hearing loss [17]. Shargorodsky et al. also found that high urinary cadmium significantly correlated with a high odds ratio for low-frequency hearing loss [18].

In the 25-year Global Burden of Disease study, hearing loss was the second most common nonfatal problem affecting the quality of life of the Chinese population [19]. The current study aims to investigate the associations between environmental cadmium and lead exposure with hearing loss in a representative sample of adults who participated in a survey of hearing loss in Zhejiang Province in China. We controlled for potential confounding factors such as demographic characteristics, lifestyle, chronic diseases, ear diseases, and working noise.

## Materials and methods

### Participants

Previously, a large study population was enrolled using a multistage stratified cluster random sampling method in Zhejiang Province from 2016 to 2018. Six cities (Hangzhou, Jiangshan, Tonglu, Jiaxing, Anji, and Jinyun) were randomly selected by stratifying according to geographical distribution and city sizes, based on the national nutrition and health survey in Zhejiang in 2012. Then, one healthcare center was randomly selected in each city, and with the coordination of local medical staff, all the participants in each healthcare center were recruited and selected by cluster sampling if they met the inclusion criteria: 1) local residents (living in the area for more than 12 months); 2) workplace noise exposure that did not meet the national noise pollution standards (less than 85 dB); 3) no previous medical history of craniocerebral injury, history of ototoxic drugs or detonation deafness; and (4) no history of inflammation or fever within 30 days before the hearing test. From the large study population, 2016 participants (1008 pairs) were selected for the present case-control study with a 1:1 paired design, and the matching criteria included age (within one year), sex, and residence cities. In the case group, participants had hearing loss while in the control group participants had normal hearing.

### Audiometric examination

Audiometry examinations were performed by trained technicians in a sound-proof chamber with noise levels below 30 dB. The devices utilized in this research were an audiometer (AT235, Interacoustics AS, Assens, Denmark) and standard headphones (TDH-39, Telephonics Corporation, Farmingdale, USA). Pure-tone air conduction hearing thresholds were tested in both ears of the participants at frequencies of 0.5, 1, 2, and 4 kHz over an intensity range of -10 to 110 dB. In the audiometric examination, participants who did not respond at least once were considered nonresponsive. To measure the reliability of participants’ responses, the 1 kHz frequency was tested twice in either ear. It was considered an unreliable response if the results differed by more than 10 dB. Finally, we computed the pure-tone average (PTA) at frequencies of 0.5, 1, 2 and 4 kHz, and hearing loss was defined as PTA >25 dB (HL) in either ear [20].

### Measurement of lead and cadmium in blood

Blood samples for lead and cadmium measurements were collected into trace-metal-free ethylenediaminetetraacetic acid tubes, and specimens were immediately transferred at 2–8°C to a central laboratory for analysis (Medical School, Hangzhou Normal University, Hangzhou, China). Lead and cadmium concentrations in venous whole blood were measured using graphite furnace atomic absorption spectrometry (Model BH2200S analyzer, BOHUI, China). For internal quality assurance and control, commercial standard reference materials (Whole Blood Lead Control (nos. 20162400100) and Cadmium Control (nos. 20162400101)) were obtained from Beijing BOHUI Innovation Biotechnology Co., Ltd. Beijing, China. Interassay coefficients of variation ranged from 2.23% to 5.98% for blood lead quality control pools and from 3.45% to 6.07% for blood cadmium quality control pools.

### Questionnaire survey

A pilot survey was carried out with an initial version of the questionnaire for validation and improvement, and then the validated questionnaire was used for the formal survey. Prior to the questionnaire survey, investigators received professional training to ensure data collection quality. The information in the questionnaire included demographic information (sex, age, education level, marital status, income level, and residence area), chronic diseases (hypertension, diabetes, hyperlipidemia, otitis media, migraine, and anemia), lifestyle factors (smoking, alcohol consumption, and fruit and vegetable intake), and environmental noise (workplace noise and recreational noise). Participants were classified as never smokers, secondhand smokers, former smokers, or current smokers. Alcohol consumption was classified as never, former, and current. Hypertension, diabetes, hyperlipidemia, otitis media, migraine, and anemia were classified based on a self-reported physician diagnosis.

If a participant indicated exposure to loud noise in the workplace at least once a week, then the participant was considered to have workplace noise exposure. If the participant had been exposed to loud noise outside of work (e.g., loud music or power tools) at least once a week, then the participants were considered to have been exposed to recreational noise. To emphasize an important point, the volume of the noise was the subjective feeling of the participant, so if a participant felt that the sound was too loud to feel uncomfortable, then he/she was considered to have been exposed to loud noise. [21]

### Data analysis

The database was established using Epidata 3.0, and analyses were performed using SPSS 22.0 for Windows (SPSS Inc., Chicago, IL, USA). A paired t-test was performed to compare the differences in the means of age, hearing threshold, and concentration of logarithmic transformed lead and cadmium levels between the case and control groups. Paired chi-square tests were used to analyze the distribution of sex, marital status, educational background, household monthly income, smoking, alcohol consumption, fruit and vegetable intake, environmental noise, chronic diseases and ear diseases between the case and control groups. Because the blood lead and cadmium contents were not normally distributed, logarithmic transformation was performed to normalize their distributions. Multivariate conditional logistic regression was used to analyze the relationship of lead, cadmium and other variables with hearing loss. Multiple linear regression was conducted to determine how all of the variables affect blood cadmium concentration.

### Ethical approval

All subjects gave their informed consent for inclusion before they participated in the study. The study was conducted in accordance with the Declaration of Helsinki, and the protocol was approved by the Ethics Committee of Hangzhou Normal University (No. 2017LL107).

## Results

The differences in the participants’ characteristics between the case group and control group are illustrated in Table 1. Due to the case and control matched design, sex composition, age distribution, and residence cities showed no difference between the case group and control group. There were 528 men and 480 women in both groups. The participants’ ages ranged from 21–89 years (52.41±11.58 in the case group and 52.38±11.60 in the control group). Regarding demographic characteristics, the difference in marital status between the groups was not statistically significant (P>0.05). However, educational level and household monthly income showed statistically significant differences (P<0.05).

**Table 1:** Differences in hearing loss status according to different variables.

Variables	N	Case n (%)	Control n (%)	p-Value
Sex				
Male	1056	528 (52.4)	528 (52.4)	1.000^a^
Female	960	480 (47.6)	480 (47.6)	
Age (year, mean ± SE)		52.41±11.58	52.38±11.60	0.067^b^
25–44	463	232 (23.0)	231 (22.9)	
44–59	989	494 (49.0)	495 (49.1)	
≧60	564	282 (28.0)	282 (28.0)	
Lead (logarithmic transformed)	2016	1.58±0.17 (μg/dL)	1.57±0.16 (μg/dL)	0.408 ^b^
Cadmium (logarithmic transformed)	2016	0.34±0.24 (μg/L)	0.32±0.22 (μg/L)	0.014 ^b^
Pure-tone average	2016	38.21±12.91 (dB)	18.68±4.93 (dB)	<0.001 ^b^
Marital status				
Unmarried	47	23 (2.3)	24 (2.5)	
Married	1840	920 (93.2)	920 (94.3)	0.396^a^
Divorced or widowed	76	44 (4.5)	32 (3.3)	
Education				
<High school	759	412 (42.0)	347 (35.9)	
High school	545	267 (27.2)	278 (28.8)	0.018^a^
>High school	643	302 (30.8)	341 (35.3)	
Household monthly income (yuan)				
<4000	712	384 (41.1)	328 (34.7)	
4000–6000	741	350 (37.5)	391 (41.4)	0.017^a^
≧6000	426	200 (21.4)	226 (23.9)	
Cities				
Jiangshan	352	176 (17.5)	176 (17.5)	
Tonglu	444	222 (22.0)	222 (22.0)	
Jiaxing	566	283 (28.1)	283 (28.1)	1.000^a^
Hangzhou	202	101 (10.0)	101 (10.0)	
Anji	354	177 (17.6)	177 (17.6)	
Jinyun	98	49 (4.9)	49 (4.9)	
Cigarette smoking				<0.001^a^
Never	880	465 (48.1%)	415 (43.0%)	
Secondhand	466	260 (26.9%)	206 (21.3%)	
Former	108	57 (5.6%)	51 (5.3%)	
Current	478	185 (24.8%)	294 (30.4%)	
Alcohol consumption				0.020 ^a^
Never	1580	763 (77.4)	817 (81.9)	
Former	43	20 (2.0)	23 (2.3)	
Current	360	203 (20.6)	157 (15.8)	
Daily fruit and vegetable intake				<0.001 ^a^
<500 g	836	485 (48.2)	351 (35.0)	
≥500 g	1174	521 (51.8)	653 (65.0)	
Workplace noise				<0.001 ^a^
None or very little	1203	553 (55.5)	650 (65.0)	
At least once a week	560	286 (28.7)	274 (27.4)	
At least once a day	233	157 (15.8)	76 (7.6)	
Recreational noise				0.647 ^a^
None or very little	1487	735 (73.6)	752 (75.4)	
At least once a week	334	174 (17.4)	160 (16.0)	
At least once a day	176	90 (9.0)	86 (8.6)	
Current hypertension				<0.001 ^a^
No	1504	691 (70.5)	813 (82.9)	
Yes	457	289 (29.5)	168 (17.1)	
Diabetes				<0.001 ^a^
No	1879	924 (94.3)	955 (97.4)	
Yes	81	56 (5.7)	25 (2.6)	
Hyperlipidemia				<0.001 ^a^
No	1846	906 (92.4)	940 (95.9)	
Yes	114	74 (7.6)	40 (4.1)	
Otitis media				<0.001 ^a^
No	1912	939 (95.8)	973 (99.3)	
Yes	48	41 (4.2)	7 (0.7)	
Migraine				0.033 ^a^
No	1894	938 (95.7)	956 (97.6)	
Yes	66	42 (4.3)	24 (2.4)	
Anemia				0.006 ^a^
No	1910	945 (96.4)	965 (98.5)	
Yes	50	35 (3.6)	15 (1.5)	

^a^: Paired χ^2^ test;

^b^: t test

The logarithmic-transformed levels of blood lead showed no significant differences between the case group (1.58±0.17 μg/dL) and control group (1.57±0.16 μg/dL). The logarithmic-transformed levels of blood cadmium were significantly higher in the case group (0.34 ± 0.24 μg/L) than the control group (0.32 ± 0.22 μg/L) (P <0.05). The PTAs of hearing thresholds were significantly higher in the case group (38.21±12.91 dB) than in the control group (18.68±4.93 dB).

Between the case group and control group, participants showed significant differences in lifestyle-related factors such as smoking, alcohol consumption, and daily fruit and vegetable intake. Regarding disease-related factors, hypertension, hyperlipidemia, diabetes, otitis media, migraine, and anemia were significantly associated with hearing loss. We also analyzed the correlation between environmental noise and hearing loss, and the results showed that workplace noise was significantly associated with hearing loss, but recreational noise was not.

The associations of participants’ blood cadmium and lead levels with hearing loss in all models are displayed in Table 2. We used conditional logistic regression to analyze the influence of blood lead and cadmium on hearing loss in distinct covariate-adjusted models. Associations of blood lead and cadmium levels with hearing loss were analyzed after adjusting for income, education, hypertension, diabetes, hyperlipidemia, otitis media, migraine, anemia, smoking, alcohol consumption, and daily fruit and vegetable intake (Model A). The association between blood cadmium level and hearing loss was statistically significant. Compared with the lowest quartile of blood cadmium concentration, the adjusted odds ratio for hearing loss was elevated in blood cadmium quartile 2 (1.869, 95% CI: 1.320–2.646), quartile 3 (2.086, 95% CI: 1.467–2.968) and quartile 4 (1.490, 95% CI: 1.048–2.119) (P-trend<0.001). After further adjusting for workplace noise exposure (Model B), the association between blood cadmium and hearing loss was still statistically significant, and the adjusted odds ratio for hearing loss was also elevated in blood cadmium quartile 2 (1.932, 95% CI: 1.356–2.751), quartile 3 (2.036, 95% CI: 1.423–2.914) and quartile 4 (1.495, 95% CI: 1.048–2.133) (P-trend<0.001). Notably, blood lead showed no association with hearing loss before or after adjusting for workplace noise exposure (Model A and Model B; P-trends>0.05).

**Table 2:** ORs (95% CIs) for hearing loss by blood cadmium and lead levels (n = 2016).

Variables		OR (95% CI)	
	N	Model A	Model B
Lead quartile (μg/dL)			
Q1 (1.50–2.66)	250/504	1	1
Q2 (2.67–3.44)	244/504	1.166 (0.832,1.635)	1.135 (0.806,1.599)
Q3 (3.45–4.70)	248/504	1.069 (0.754,1.515)	1.038 (0.731,1.475)
Q4 (4.71–16.50)	266/504	1.036 (0.716,1.498)	1.016 (0.700,1.475)
*p*-trend		0.992	0.900
Cadmium quartile (μg/L)			
Q1 (0.00–0.53)	224/504	1	1
Q2 (0.54–0.92)	270/515	1.869 (1.320,2.646)	1.932 (1.356,2.751)
Q3 (0.93–1.62)	267/497	2.086 (1.467,2.968)	2.036 (1.423,2.914)
Q4 (1.63–57.81)	247/500	1.490 (1.048,2.119)	1.495 (1.048,2.133)
*p*-trend		<0.001	<0.001

Model A is adjusted for income level, education level, hypertension, diabetes, hyperlipidemia, acute and chronic otitis media, migraine, anemia, smoking, alcohol consumption, and fruit and vegetable intake.

Model B is further adjusted for workplace noise.

The influencing factors of blood cadmium content in Chinese residents were also analyzed. As shown in Table 3, the results of multiple linear regression analyses showed that young age (less than 60 years), male sex and current smoking were associated with increased blood cadmium concentration. Additionally, a positive association between blood cadmium and lead concentrations was found. Current smoking and lead exposure were more related to blood cadmium than other factors.

**Table 3:** Analysis of the influencing factors of blood cadmium.

Variables	B	*p*-Value
Age	-0.064	<0.001
Sex		
Male	Reference	
Female	-0.089	0.002
Monthly income (yuan)		
≤4000	Reference	
4001–6000	-0.014	0.650
≥6001	0.030	0.345
Education		
<High school	Reference	
High school	0.024	0.414
>High school	0.035	0.302
Cigarette smoking		
Never	Reference	
Secondhand	-0.078	0.003
Former	0.027	0.296
Current	0.122	<0.001
Alcohol consumption		
Never	Reference	
Former	-0.027	0.259
Current	-0.047	0.087
Daily fruit and vegetable intake		
<500 g	Reference	
≥500 g	-0.009	0.718
Lead concentration	0.113	<0.001

* B is the standardized regression coefficient

## Discussion

Many experimental studies have shown the harmful effects of lead and cadmium on hearing functions. The cochlear component of hearing is more vulnerable to cadmium toxicity than other parts of the auditory system [13]. Cadmium produces reactive oxygen species, leading to mitochondrial membrane depolarization, cell apoptosis and cysteine protease activation and increasing extracellular signal-regulated kinase activation [13, 22]. Cumulative lead exposure may lead to the death of auditory hair cells [20] or can alter the structure and functions of axons in the auditory nucleus of the brainstem [12], eventually leading to impairment of sound conduction [23].

After adjusting for other variables, we did not find an association between blood lead and hearing loss. In the present study, the lead concentration ranged from 1.50 to 16.50 μg/dL. Similarly, in 2,535 American adolescents, Josef et al. found that blood lead exposure was not associated with low-frequency hearing loss (0.5, 1 and 2 kHz), and the lead concentration range was within 2 μg/dL [18]. Choi et al. also did not find an association between blood lead and hearing loss in Korean adults, and the lead concentration ranged from 0.33 to 26.51 μg/dL [20]. The Occupational Safety and Health Administration safety standard is currently 38.6 μg/dL for lead in the whole blood (Agency for Toxic Substances and Disease Registry reported by Choi et al. (2012) [17]. Choi et al. conducted another study in American adults; the lead concentration ranged from 0.20–54.00 μg/dL, and the lead level was found to be positively associated with hearing loss [17]. We hypothesize that it is likely that the blood lead concentration in our study was not high enough to trigger detectable ototoxic effects.

Our findings showed that increased blood cadmium could elevate the risk for hearing loss in Chinese adults. Compared with the first quartile of blood cadmium, the risk for hearing loss increased in the second and third quartiles but decreased in the fourth quartile, showing an inverse U pattern. This result is different from the findings of previous studies; Choi et al. also found a positive relationship of blood cadmium with hearing loss in the American general population, but the risk for hearing loss was consistently increased with the increase in cadmium concentration [17]. After analysis, we found that in the research done by Choi et al. approximately 0.11% of the participants exceeded the cadmium limits (5 μg/L) [17]. In our study, 5.8% of the participants (n = 166) exceeded the limits, and all of them were in the highest quartile. We hypothesize that there are some other underlying confounding factors that cannot be explained by our model that induced the differences, which needs further investigation in the future. Additionally, Josef et al. found that increased urinary cadmium exposure was associated with an elevated risk of low-frequency hearing loss in American adolescents [18].

In this research, current smoking and lead exposure showed an association with blood cadmium. Cadmium typically enters the body from contaminated food, such as vegetables, grain, and shellfish; water; polluted air, and pollutants emitted during metal smelting and refining, and the production of batteries, alloys, pigments and plastic stabilizers [7, 24]. Similarly, environmental lead pollutants mainly result from battery manufacturing, steel welding or cutting operations, construction, gasoline and paint [9]. This probably explains the significant association of lead with blood cadmium in our research.

In China, the number of smokers exceeds 300 million, and 740 million nonsmokers are exposed to secondhand smoke [8]. In our research, 54.45% of the participants were secondhand smokers, former smokers or current smokers. Moreover, cigarette smoking itself has been prevalently verified to be a risk factor for hearing loss in cross-sectional studies or cohort studies [25]. The carbon monoxide released from cigarette smoke is considered a potential ototoxin that can shift the hearing threshold [26]. Additionally, cigarette smoke is a resource of cadmium pollution [7, 27], and its ototoxic effect is probably attributed to cadmium to some extent, which can be supported by our results.

The current study has several strengths. Above all, this study first examined the relationship between blood lead and cadmium levels and hearing loss in a large general Chinese population, rather than focusing on occupational workers or animals. Second, we performed case-control analysis matched for age, sex and residence cities, which can exclude the influences of these factors. For the nonoccupational population, age proved to be the main influential factor for hearing loss [28]. Third, the present research adjusted for many potential key confounding factors that have been shown to be associated with hearing loss, such as workplace noise exposure, smoking, hypertension, diabetes, and ear diseases [29, 30, 31].

Nonetheless, this study had some limitations. This was a case-control study, and causal relationships between heavy metals and hearing loss cannot be inferred. Second, occupational noise and recreational noise information were obtained from self-reporting, which probably caused potential misclassification. Third, pure-tone air conduction hearing threshold testing was used for hearing loss detection, but some hearing loss caused by nerve conduction latency problems adversely affected by lead and cadmium exposure may need to be detected using a more sensitive method, such as auditory brainstem response (ABR) testing. Last, propensity score matching is a great method for decreasing bias, and this case-control study would have been better if the controls and the cases were matched with this method to include additional factors, such as noise exposure.

## Conclusions

In summary, the present analysis of a well-defined representative sample of Chinese adults found a statistically significant association between current exposure to environmental cadmium and the risk of hearing loss, independent of known risk factors including various types of noise exposures or clinical risk factors. However, we found no significant association between environmental lead exposure and hearing loss in adults. Our findings indicate that knowledge of cadmium exposure may allow for earlier interventions in the public health community and that the government should make efforts to reduce environmental lead and cadmium exposures and conduct interventions for related lifestyles, such as tobacco control, to prevent hearing loss in the general population. Further studies with prospective designs and wide ranges of exposure are needed to confirm the results due to concerns related to causal inferences and potential reverse causality.

## Supporting information

S1 Table ORs (95% CIs) for hearing loss according to different variables (n = 2016). (DOCX)

S1 File Dataset of this study. (SAV)
