# Supplementary material for: Environmental exposure to lead and cadmium and hearing loss in Chinese adults: A case-control study
Source: PLoS One. 2020 May 20;15(5):e0233165. doi: 10.1371/journal.pone.0233165 (PMC7239478; doi:10.1371/journal.pone.0233165)
Supplement: S1 Table — (DOCX) [file pone.0233165.s001.docx]

**Table S1 ORs (95% CIs) for hearing loss by different variables (n=2016)**

| **Variables** | **OR (95%CI)** | |
| --- | --- | --- |
| Lead quartile (µg/dL) | Model A | Model B |
| Q_1_（1.50-2.66） | 1 | 1 |
| Q_2_（2.67-3.44） | 1.166(0.832,1.635) | 1.135(0.806,1.599) |
| Q_3_（3.45-4.70） | 1.069(0.754,1.515) | 1.038(0.731,1.475) |
| Q_4_（4.71-16.50） | 1.036(0.716,1.498) | 1.016(0.700,1.475) |
| *p*-trend | 0.992 | 0.900 |
| Cadmium quartile (µg/L) |  |  |
| Q1（0.00-0.53） | 1 | 1 |
| Q2（0.54-0.92） | 1.869(1.320,2.646) | 1.932(1.356,2.751) |
| Q3（0.93-1.62） | 2.086(1.467,2.968) | 2.036(1.423,2.914) |
| Q4（1.63-57.81） | 1.490(1.048,2.119) | 1.495(1.048,2.133) |
| \| *p*-trend \|  \|  \| \| --- \| --- \| --- \| | 0.000 | 0.000 |
| Current Hypertension |  |  |
| No | 1 | 1 |
| Yes | 1.758 (1.306,2.367) | 1.772 (1.306,2.406) |
| Otitis media |  |  |
| No | 1 | 1 |
| Yes | 8.361(2.388,29.271) | 12.863(2.864,57.769) |
| Household Monthly Income (yuan) |  |  |
| <4000 | 1 | 1 |
| 4000-6000 | 0.685(0.490,0.957) | 0.740(0.527,1.040) |
| ≧6000 | 0.633(0.417,0.962) | 0.649(0.424,0.995) |
| Cigarette smoking |  |  |
| Never smoker | 1 | 1 |
| Secondhand smoker | 1.533(1.096,2.151) | 1.445(1.025,2.038) |
| Formal smoker | 2.171(1.177,4.002) | 2.097(1.128,3.897) |
| Current smoker | 2.104(1.446,3.062) | 1.936(1.324,2.830) |
| Daily fruit and vegetable intake |  |  |
| <500g | 1 | 1 |
| ≥500g | 0.526(0.405,0.682) | 0.552(0.422,0.720) |
| Workplace noise |  |  |
| No or very little | — | 1 |
| At least once a week | — | 1.061(0.804,1.401) |
| At least once a day | — | 1.655(1.096,2.499) |

Note: Model A adjusts income level, education level, hypertension, diabetes, hyperlipidemia, acute and chronic otitis media, migraine, anemia, smoking, drinking, fruits and vegetables.

Note: Model B further adjusts the workplace noise based on Model A.
